# Medicines without Doctors: In Mozambique, Salaries Are Not the Biggest Problem

**DOI:** 10.1371/journal.pmed.0040236

**Published:** 2007-07-31

**Authors:** Wouter Arrazola de Oñate

As a former collaborator of the Faculty of Medicine of the Mozambican Eduardo Mondlane University, I have been screaming the same message as Ooms and collegues [1] for years now. I am glad that finally some attention has been given to this fact.

Although I do agree with the general message of the article, in the case of Mozambique, the brain drain is not the biggest problem. Neither are the salaries. It's the pure lack of doctors. Only up to 60 doctors a year are trained at the University for a population of 18 million. In 2004, Mozambique had about 700 medical doctors, expatriates from all nongovernmental organizations and projects included. I do not agree that increasing salaries would be the most efficient solution. This will not create more doctors. This will create salaries without doctors.

The Mozambican Ministry of Health received hundred of millions of dollars from international donors for the National AIDS Plan, while the Faculty of Medicine, dependant on the Ministry of Education, was struggling to survive. Not a single dollar from all the AIDS millions went to support the basic education of doctors. Not out of bad will, but because donors have too many restrictions.

Doctors are not only needed for the HIV/AIDS epidemic—they also treat the many cases of malaria, tuberculosis, diarrhea in children, leishmaniasis, fistula, sexually transmitted infections… They perform caesarean sections and other life-saving surgeries.

It's my strong opinion that direct investment in training of medical doctors is the most effective sector-wide approach in public health. It is the most obvious and well-defined contribution to the health-related Millennium Development Goals and development in general. I do not understand why so few donors agree with this.

I can only speak for the case of Mozambique.
